# Effect of polymicrobial sepsis on the respiratory mechanism of rats previously exposed to cigarette smoking

**DOI:** 10.1186/cc12984

**Published:** 2013-11-05

**Authors:** Glauber C Lima, Kalynca KV Aragão, Viviane G Portella, Lúcia RL Diniz, Sales A Cavacante, Daniel S Serra, Andrelina N Coelho-de-Souza

**Affiliations:** 1 Superior Institute of Biomedical Sciences, State University of Ceará, Fortaleza, CE, Brazil; 2 Faculty of Medicine, UniChristus, Fortaleza, CE, Brazil; 3 Center of Science and Technology, State University of Ceará, Fortaleza, CE, Brazil

## Background

The objective was to evaluate the profile of respiratory mechanism of septic female rats previously submitted to exposure of cigarette smoking.

## Materials and methods

Initially, female rats (230 to 300 g) were randomly divided into a control group (NS) kept with no manipulation and a cigarette smoking-induced respiratory disorders group (S). A rat model used to induce respiratory disorders was established by exposure to cigarette smoking (8 units/15 minutes) daily for 6 weeks. Twenty-four hours after the last cigarette smoking exposure session, each group underwent cecal ligation and puncture procedures to induce polymicrobial sepsis (CLP group) or only underwent a laparotomy (sham group), resulting in the following four experimental groups: Sham-NS (*n *= 11), Sham-S (*n *= 11), CLP-NS (*n *= 6) and CLP-S (*n *= 9). The profile of respiratory mechanism was evaluated by forced oscillation measurements using a computer-controlled piston ventilator (flexiVent; SCIREQ Inc.) at 24 hours CLP or Sham procedures. The respiratory system parameters evaluated were calculated in flexiWare7 software. All experimental procedures used in our study were approved by the Institutional Animal Ethics Committee (nº 11221971-3/47).

## Results

Among the experimental groups, no significant difference in airway resistance was verified, while prior exposure to cigarette smoking decreased the tissue resistance of sham-operated rats (0.77 ± 0.03 vs. 0.55 ± 0.01 cmH_2_O.second/ml, Sham-NF and Sham-F, respectively) as well as inhibiting the increase in tissue resistance induced by sepsis (1.11 ± 0.11 vs. 0.76 ± 0.03 cmH_2_O.second/ml, CLP-NS and CLP-S, respectively). The prior exposure to cigarette smoking did not alter the lung compliance of sham-operated rats, but it blocked the CLP-induced reduction of lung compliance (0.82 ± 0.04, 0.21 ± 0.11 and 0.57 ± 0.07 cmH_2_O.second/ml, Sham-NS, CLP-NS and CLP-S, respectively). Similarly, cigarette smoking blocked the CLP-induced decrease of inspiratory capacity (7.85 ± 0.25, 4.96 ± 1.49 and 7.00 ± 0.41 cmH_2_O.second/ml, Sham-NS, CLP-NS and CLP-S, respectively) but did not alter the inspiratory capacity from sham-operated rats (8.68 ± 0.2 cmH_2_O.second/ml, Sham-S) compared with Sham-NS.

## Conclusions

In contrast to sham-operated rats, cigarette smoking inhibited changes in the resistance, compliance and inspiratory capacity of the respiratory system of CLP-operated rats.

